# A Rare Case of Non-small Cell Lung Carcinoma Squamous Phenotype with Epstein-Barr Virus Positivity with Prolonged Response to both Chemotherapy and Radiotherapy

**DOI:** 10.3779/j.issn.1009-3419.2021.101.15

**Published:** 2021-07-20

**Authors:** CarolinaNavarro RODRIGUEZ, MuhammadShahid IQBAL, Max ROBINSON, Graham BURNS, Alastair GREYSTOKE

**Affiliations:** 1 Department of Clinical Oncology, Northern Centre for Cancer Care, The Newcastle-upon-Tyne Hospitals NHS Foundation Trust, Newcastle-upon-Tyne, UK; 2 Department of Pathology, Northern Centre for Cancer Care, Freeman Hospital, The Newcastle-upon-Tyne Hospitals NHS Foundation Trust, Newcastle-upon-Tyne, UK; 3 Department of Respiratory Medicine, Royal Victoria Infirmary, Northern Centre for Cancer Care, The Newcastle-upon-Tyne Hospitals NHS Foundation Trust, Newcastle-upon-Tyne, UK; 4 Department of Medical Oncology, Northern Centre for Cancer Care, The Newcastle-upon-Tyne Hospitals NHS Foundation Trust, Newcastle-upon-Tyne, UK

**Keywords:** Lung neoplasms, Epstein-Barr Virus, Tuberculosis, Chemotherapy, Radiotherapy

## Abstract

We present a rare challenging case of metastatic non-small cell lung cancer with Epstein-Barr virus positivity that was also diagnosed with pulmonary tuberculosis at the same time. Palliative chemotherapy gemcitabine and carboplatin was started after two weeks of anti-tuberculosis treatment with the hopes that this period would be sufficient to keep acid fast bacilli non-viable to minimise risk of tuberculosis re-activation due to chemotherapy induced immunosuppression. She completed four cycles of chemotherapy and six months of anti-tuberculosis treatment with good results and minimal side effects. Two years later, there was disease recurrence in cervical and mediastinal lymph nodes which was treated with local treatment i.e. surgery and palliative radiotherapy. It has been two years since last radiotherapy and overall more than five years since diagnosis with no active disease at present. Given the complexity and rarity of this case, significant multidisciplinary team involvement, including oncologists and radiation oncologists, pulmonologists with special interest in tuberculosis and pathologists was necessary throughout.

## Introduction

The Epstein-Barr virus (EBV) is associated with a variety of lymphoproliferative and neoplastic diseases^[1]^. The possible aetiology link of EBV with development of lung cancer remains controversial. There are a few laboratory-based studies postulating the possible link of EBV with non-small cell lung cancer (NSCLC)^[2]^ however other studies did not find any causative link^[3, 4]^. In addition, there are numerous studies conducted on the role of tuberculosis (TB) and lung cancer as chronic inflammation and fibrotic changes related to TB can lead to genetic mutations and alterations^[5]^.

## Case presentation

A 49-year-old woman presented with 6-months history of weight loss and intermittent cough which did not respond to antibiotics or inhalers. She never experienced haemoptysis. Diagnostic workup led to diagnosis of T4N3M1b squamous cell carcinoma (SCC), epidermal growth factor receptor (*EGFR*) and anaplastic lymphoma kinase (*ALK*) mutations negative. Endobronchial biopsy also showed caseating granulomas indicating active mycobacterium TB infection. She was started promptly on anti-TB medications and was referred for anti-cancer management.

Her past medical history included, SLE with developing Jaccoud's arthropathy, scleritis and urticarial vasculitis.

She was lifelong non-smoker and non-drinker, who had moved from Hong Kong to the UK in late childhood.

**Figure 1:**
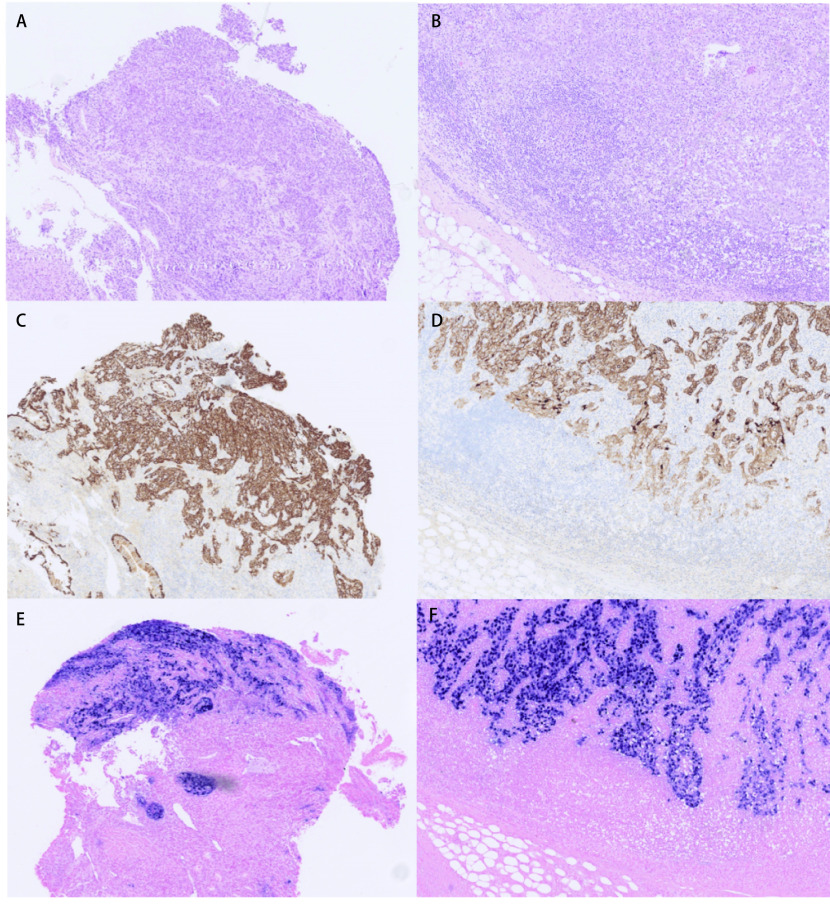
Photomicrographs showing an Epstein-Barr virus (EBV) positive squamous cell carcinoma of the lung that metastasized to lymph nodes in the neck (Original magnification ×100). The lung biopsy contained a poorly differentiated squamous cell carcinoma (A), that expressed cytokeratins 5 and 6 (C) and showed evidence of EBV infection by EBV-encoded RNA (EBER) *in situ* hybridisation (E). The corresponding cervical lymph node metastasis showed an identical profile (B, D, F).

Left neck node excisional biopsy confirmed metastatic SCC strongly positive for EBV infection with EBV-encoded RNA-1 (EBER1) *in-situ* hybridisation (Fig 1). There was no abnormality in the nasopharynx. The tumour was tested on the Cancer Research UK stratified medicine programme^[6]^ and unusually analysis showed no abnormality in the 28 genes tested including those commonly mutated in squamous cancer such as *PI3K* and *FGFR*. She was started on gemcitabine and carboplatin chemotherapy in conjunction with appropriate TB treatment and completed four cycles with excellent response.

She tolerated her TB treatment remarkably well despite it being in conjunction with chemotherapy. Thrombocytopenia was the only chemotherapy related side effect she experienced in her last two cycles requiring platelet transfusions.

Twenty five months after, there was recurrence in right supraclavicular fossa (treated with neck dissection) and paratracheal region (treated with radiotherapy 30 Gy in 10 fractions). Six months later, there was disease recurrence in her right neck which was treated with further radiotherapy. It has been more than 25 months since last radiotherapy with latest imaging showing no evidence of active disease.

## Discussion

Published in 1998, Chen *et al*^[7]^ reported a study involving 127 cases of NSCLC in Taiwan population. EBV positivity was investigated using EBV-encoded RNA-1 (EBER1) transcripts by *in situ* hybridization and the expression of latent membrane protein-1 (LMP-1) and bcl-2 protein by immunohistochemistry. EBER1 was detected in 11 cases: 6 SCC and 5 lymphoepithelioma-like carcinoma (LEC). Five of the 6 EBV positive SCC showed diffuse but weak staining. Three of the 6 patients with the EBER-1 positive SCC were smokers. Although EBER-1 positive LEC patients were with younger age and a better 2-year survival, this difference was not observed in EBER-1 positive SCC patients. Overall, the presence of EBER-1 transcript showed no correlation with the 2-years survival rate (*P* > 0.05).

In relation to TB, Keikha *et al*^[5]^ suggested that TB related chronic inflammation and fibrosis can induce genetic mutations and alternations. Increasing rates of interleukin (IL)-17 and tumor necrosis factor alpha (TNF-α) as a result of necrosis, apoptosis or TB reactivation, will either decrease P53 activity or increase the expression of B-cell lymphoma-2 (Bcl-2), decrease Bax-T, and cause the inhibition of caspase-3 expression due to decreasing the expression of mitochondria cytochrome oxidase. There are multiple studies being conducted regarding the possible association between TB and risk of lung cancer. Liang *et al*^[8]^ performed a systematic review of 37 case-control, 4 cohort studies and a meta-analysis of risk estimates where a significant association between TB and adenocarcinoma was found but no significant associations with squamous and small cell type of lung cancer were observed.

Hirashima *et al*^[9]^ concluded that concurrent chemotherapy and TB treatment is effective and safe for treating patients with active TB, following a restrospective study of 30 patients with different types of malignancies and active TB.

Apart from rarity of tumour type, this case was of interest due to the unique challenge of treating a dual pathology. There was equal urgency to commence TB treatment and chemotherapy. A clinical decision had to be made with a paucity of supporting published data to inform that decision and a multidisciplinary approach, involving oncologists, radiation oncologists, pulmonologists with especial interest in TB and pathologists was required in order to achieve the best outcome. Multidisciplinary treatment is essential in lung cancer to optimise the survival and quality of life of these patients^[10]^.

## Conclusion

This case in a woman who was brought up in Hong Kong fits with above literature suggesting that this cancer is more common in the Eastern Populations. Similar to EBV driven nasopharyngeal cancer, the cancer in this case was very sensitive to both chemotherapy and radiotherapy. This diagnosis should be considered in patients with squamous lung cancer who live or have lived in the Far East particularly in the setting of unusual clinical factors such as light smoking history, lack of classical molecular abnormalities on sequencing or deep response to chemotherapy.
